# In search of tail-anchored protein machinery in plants: reevaluating the role of arsenite transporters

**DOI:** 10.1038/srep46022

**Published:** 2017-04-06

**Authors:** Manuel Maestre-Reyna, Shu-Mei Wu, Yu-Ching Chang, Chi-Chih Chen, Alvaro Maestre-Reyna, Andrew H.-J. Wang, Hsin-Yang Chang

**Affiliations:** 1 Institute of Biological Chemistry, Academia Sinica, Taipei, Taiwan; 2 Department of Marine Biotechnology and Resources, National Sun Yat-sen University, Kaohsiung, Taiwan; 3 Doctoral Degree Program in Marine Biotechnology, National Sun Yat-Sen University, 70 Lien-Hai Road, Kaohsiung 80424, Taiwan; 4 Doctoral Degree Program in Marine Biotechnology, Academia Sinica, 128 Academia Road, Section 2, Nankang, Taipei 11529, Taiwan; 5 Escuela Tecnica Superior de Ingenierios Industriales, Universidad Politecnica de Valencia, Valencia, Spain; 6 Core Facilities for Protein Structural Analysis, Academia Sinica, Taipei, Taiwan; 7 PhD Program for Translational Medicine, College of Medical Science and Technology, Taipei Medical University, Taipei, Taiwan; 8 The Asia-Pacific Ocean Research Center, National Sun Yat-sen University, Kaohsiung 804, Taiwan

## Abstract

Although the mechanisms underlying selective targeting of tail-anchored (TA) membrane proteins are well established in mammalian and yeast cells, little is known about their role in mediating intracellular membrane trafficking in plant cells. However, a recent study suggested that, in green algae, arsenite transporters located in the cytosol (ArsA1 and ArsA2) control the insertion of TA proteins into the membrane-bound organelles. In the present work, we overproduced and purified these hydrophilic proteins to near homogeneity. The analysis of their catalytic properties clearly demonstrates that *C. reinhardtii* ArsA proteins exhibit oxyanion-independent ATPase activity, as neither arsenite nor antimonite showed strong effects. Co-expression of ArsA proteins with TA-transmembrane regions showed not only that the former interact with the latter, but that ArsA1 does not share the same ligand specificity as ArsA2. Together with a structural model and molecular dynamics simulations, we propose that *C. reinhadtii* ArsA proteins are not arsenite transporters, but a TA-protein targeting factor. Further, we propose that ArsA targeting specificity is achieved at the ligand level, with ArsA1 mainly carrying TA-proteins to the chloroplast, while ArsA2 to the endoplasmic reticulum.

Cells contain numerous integral membrane proteins (IMPs) that mediate a range of essential activities. In eukaryotes, most IMPs are first inserted into the endoplasmic reticulum (ER) via a co-translational pathway involving the signal recognition particle (SRP)^1^,^2^,^3^. However, nearly 5% of all eukaryotic IMPs are tail-anchored (TA)^4^,^5^,^6^, containing a cytosolic N-terminal domain and a single C-terminal transmembrane domain. TA proteins comprise a large family of integral membrane proteins in all living creatures. Accordingly, over fifty TA proteins are predicted to be expressed in yeast^4^, around ten in prokaryotes^5^, while more than 400 are predicted in mammals^6^, and plants^7^. In eukaryotes, these proteins are found on most cellular and organelle membranes, where they mediate a remarkably wide array of essential cellular processes. Examples of TA proteins include the soluble N-ethylmaleimide–sensitive factor attachment protein receptors (SNAREs) mediating vesicular trafficking and fusion, the Bcl-2 family involved in the regulation of apoptosis, and the subunits of the ER, mitochondrial, and plastidial outer membrane translocons^4^,^8^,^9^,^10^,^11^,^12^,^13^.

TA proteins cannot access the co-translational membrane insertion pathway^6^, but instead utilize a novel, SRP-independent, post-translational pathway (PTP)^14^,^15^,^16^,^17^. Recently, the core machinery for the yeast and mammalian PTP has been identified, including the structures of several crucial protein complexes^18^,^19^,^20^,^21^,^22^,^23^,^24^,^25^. Here, the cytosolic ATPase TRC40 (ASNA-1 in human and Get3 in yeast) protects the TA transmembrane domain during cytosolic transport, and mediates contacts with ER-bound receptors^14^,^15^,^16^,^17^. However, relatively little is known about the molecular machinery responsible for PTP in plant cells. Although few TA proteins have been experimentally characterized in plants, they appear to play crucial roles^26^,^27^,^28^,^29^,^30^. For example, plant TA proteins appear to be involved in metabolic regulation, defense response, environmental stress response, etc. throughout plant growth and development^27^,^31^,^32^.

Recent studies on *Chlamydomonas reinhardtii* showed that ArsA1 (arsenite transporter), a protein sharing sequence homology with TRC40, may control the insertion of TA protein TOC34 (the chloroplast outer membrane translocon)^33^. Its absence leads to a small chloroplast and severely decreased chlorophyll content, implying a role in the biogenesis of nuclear-encoded chloroplast proteins^33^. ArsA proteins are bacterial two-domain ATPases composed of a single polypeptide, which act as catalytic subunits in arsenite and antimonite eflux pumps^34^,^35^. However, human TRC40/ASNA-1, was also originally annotated as an arsenic eflux pump ATPase domain, and is a member of the ArsA protein family (uniprot accession code O43681)^36^. To date, it is unclear whether algal ArsA proteins are involved in the TA-protein PTP, or provide arsenite resistance.

Here, we report the biochemical characterization of recombinant *C. reinhardtii* ArsA1 and ArsA2. Our results indicate that, similarly to TRC40, the ATPase activity of *C. reinhardtii* ArsA (Cr-ArsA) proteins is arsenite and antimonite independent. Further, we show that both proteins form a complex with a co-expressed TA protein, containing a C-terminal His-tag. Finally, three-dimensional structural modeling and molecular dynamics (MD) simulations allowed us contextualize ArsA2 behavior in light of recently published structural information regarding Get3-TA protein complexes. Thus, our data provide new insights into the molecular mechanism of TA protein recognition and chaperoning in plants, an as yet uncharacterized field.

## Results and Discussion

**Figure 1:**
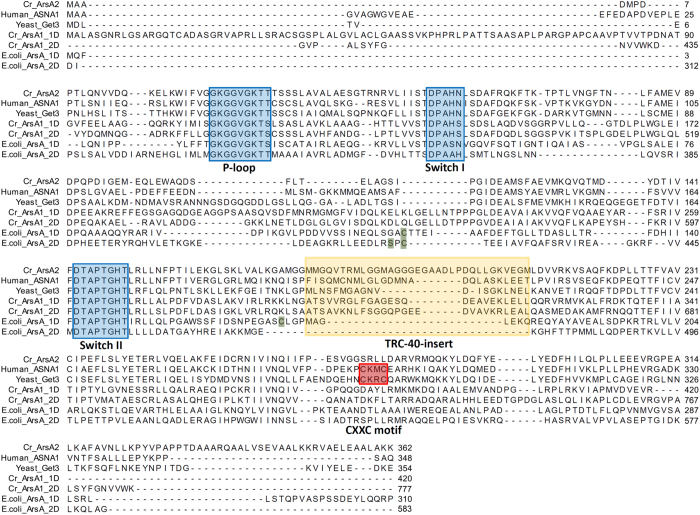
Sequence alignment of Get3 homologues and bacterial ArsA enzymes. Three conserved ATPase amino acid sequence motifs (P-loop, Switch I and Switch II, shown in blue) and the zinc-binding domain (CXXC motif, shown in red) for dimerization are highlighted. Three conserved cysteine and a serine residues (Cys-113, Cys-172, Cys-422 and Ser-420) used by bacterial ArsA to bind metalloids are colored green. The eukaryotic TRC40/Get3 homologs possess an approximate 20~30-residue insertion (TRC40-insert, shown in orange), which is absent from bacterial ArsA homologs. 1D and 2D represent the first (N-terminus) and second (C-terminus) ATPase domain in Cr-ArsA1 or *E. coli* ArsA.

**Figure 2:**
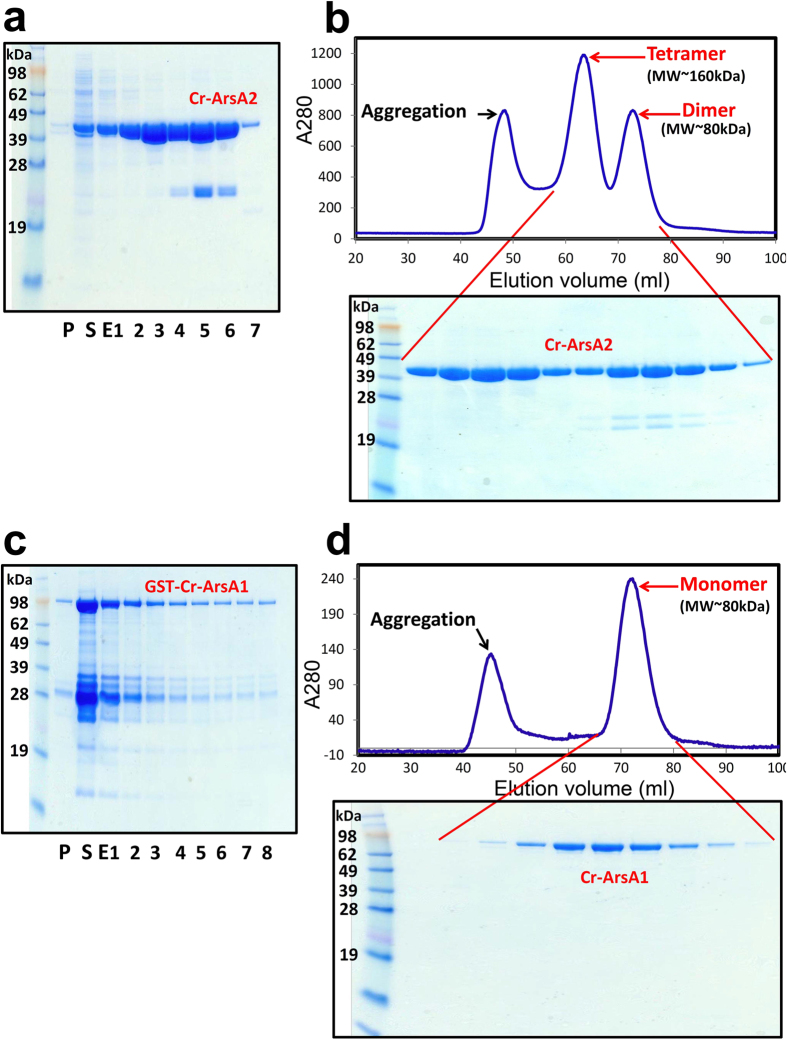
Purifying Cr-ArsA2 and Cr-ArsA1. (**a**) Soluble, high level expression of Cr-ArsA2 in *E. coli* and purification by Ni-NTA chromatography. (**b**) Fractions from preparative size exclusion chromatography. (**c**) Expression of GST-Cr-ArsA1 in *E. coli* and purification by Ni-NTA chromatography. (**d**) Cr-ArsA1 was fractionated by size exclusion chromatography after the cleavage with 6xHis-tagged TEV protease and removal of residual uncleaved GST protein and 6xHis-tagged TEV protease by subtractive Ni-NTA purification. P, pellet; S, supernatant; E, elution.

Cr-ArsA1 comprises a single polypeptide with two ATPase domains (~30% sequence identity to mammalian TRC40), whereas Cr-ArsA2 has only one ATPase domain with 40~50% sequence identity to TRC40 and Get3 (Fig. 1). A sequence alignment also reveals that both proteins contain three highly conserved, and essential regions for TRC40-like proteins, i.e. the P-loop, the switch I, and the switch II motifs^18^. Eukaryotic TRC40 enzymes also contain an approximately 20~30-residue insertion in the α-helical domain (TRC40-insert)^18^, which is absent from bacterial ArsA homologs, but found in Cr-ArsA2 as well as Cr-ArsA1 (Fig. 1). Both Cr-ArsA2 and Cr-ArsA1 lack conserved cysteine residues (Cys-113, Cys-172 and Cys-422 in *E. coli* ArsA) used by bacterial ArsA to bind metalloids^35^, and also lack the CXXC dimerization motif, generally present in eukaryotic TRC40/Get3. However, when recombinant Cr-ArsA2 was purified (Fig. 2), it formed both dimers (~80 kDa peak) and tetramers (~160 kDa peak, Fig. 2b). This is consistent with a previous study in the characterization of an archaeal Get3, which concluded that certain putative Get3 homologues may still oligomerize, even in the absence of CXXC motif^24^. As predicted by the alignment, purified Cr-ArsA1 revealed a monomeric architecture, with a molecular weight of about 80 kDa, much like bacterial ArsA proteins (Fig. 2c and d).

Another feature that helps separate TRC40-like proteins from arsenite transporters is the formers’ oxyanion-independent ATPase activity. In the absence of arsenite/antimonite, both dimeric Cr-ArsA2 and monomeric Cr-ArsA1 present a strong basal ATPase activity (400–480 nmol/min/mg), compared to *E. coli* ArsA and human ASNA-1 (0.06 and 17 nmol/min/mg), but similar to yeast Get3 (~418 nmol/min/mg, Table 1). In the presence of high oxyanion concentrations (100 mM), however, both arsenite and antimonite fail to elicit a strong effect on either algal protein (Table 1). These data are in complete contrast to previous studies in bacterial ArsA activity, which increased about 4-fold with arsenite and 32-fold with antimonite^34^.

**Figure 3:**
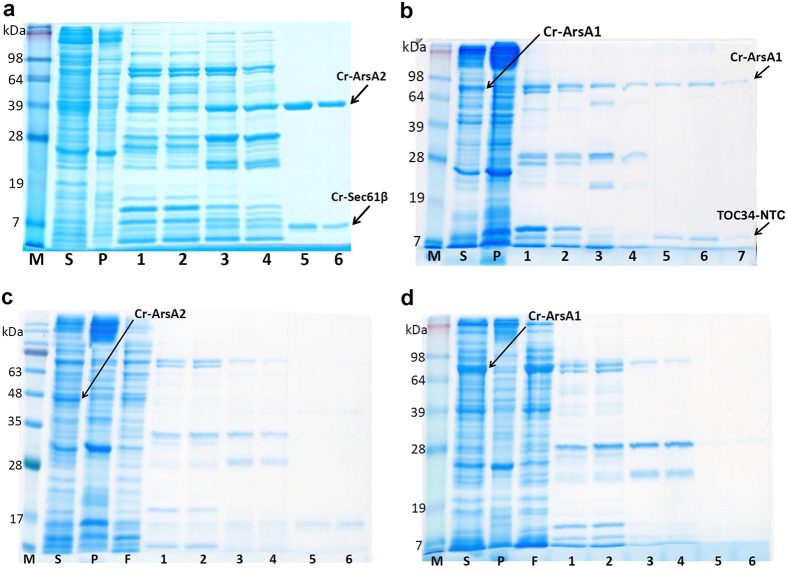
Selectivity of Cr-ArsA interactions with TA protein Substrates. (**a**) Cr-ArsA2/Cr-sec61β and (**b**) Cr-ArsA1/Cr-TOC34-NTC TA protein complex affinity purified by recombinant co-expression. Arrows indicate Cr-ArsA2 and Cr-Sec61β with a C-terminal His-tag in (**a**) panel; Cr-ArsA1 and Cr-TOC34 transmembrane domain (NTC) with a C-terminal His-tag in (**b**) panel. (**c**) The co-expression and purification of Cr-ArsA2 with Cr-TOC34-NTC containing a C-terminal His-tag, and Cr-ArsA1 with histidine-tagged Cr-sec61β in (**d**) panel. SDS-PAGE gel analysis: lane 1 and 2 – wash with 50 mM imidazole; lane 3 and 4 – wash with 100 mM imidazole; lane 5 and 6 (or 7) - elution with 250 mM imidazole. S, supernatant; P, pellet; F, flow through.

However, the most convincing evidence of TA-targeting activity by both Cr-ArsA proteins is provided by their co-expression and Ion Metal Affinity Chromatography (IMAC) co-purification with C-terminal 6xHis-tag fused TA proteins. Cr-ArsA2 readily bound both full length human Sec61β, and its *C. reinhardtii* orthologue, Cr-Sec61β (Fig. 3a and Fig. S1). Conversely, no interaction took place with TMD-deleted Sec61β (Fig. S2 a and b). On the other hand, Cr-ArsA1 binding to the Cr-TOC34-NTC domain, which contains the Cr-TOC34-TMD (Fig. 3b), (see Materials and Methods for details) was confirmed via mass spectrometry (Fig. S3). Although the C-terminal portion of both TA proteins are transmembrane helices, the presence of their corresponding ArsA protein is enough for them to be produced in the soluble fraction of the cell lysate. This behavior has also been observed for other TRC40-TA protein complexes, such as yeast Get3^19^,^37^. In order to exclude unspecific interactions between the ArsA proteins and the purification resin, we further performed negative controls, in which we passed non-tagged ArsA1 through the IMAC column (Fig. S2 c and d). As expected, the protein could not be purified in the absence of its his-tagged NTC ligand. Most interestingly, when reverse co-purification took place, i.e. Cr-ArsA1 with Cr-Sec61β, and Cr-ArsA2 with Cr-TOC34-NTC, neither the putative TA-carriers, nor the corresponding TMD/NTC-domains could be isolated, although both the carriers and substrates were co-expressed (Fig. 3c,d and Fig. S4). Indeed, to us, this clearly suggests that Cr-ArsA1 and Cr-ArsA2 have different ligand specificities, and therefore, may target different sub-cellular compartments, namely Cr-ArsA1 for chloroplast^33^, and Cr-ArsA2 for the endoplasmic reticulum^38^.

**Figure 4:**
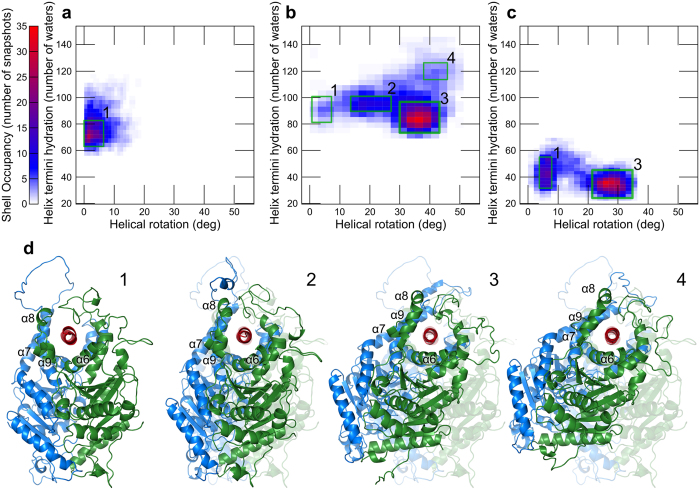
TA protein Pep12 MD simulations in complex with Get3 and Cr-ArsA2. (**a**) Heat map of the 2-D frequency analysis for the Pep12-Get3 complex. Histograms quantify the Pep12 termini hydration shell as number of waters within 5 Å of the first and last two amino-acids of the ligand. On the other hand, the rotation of Get3 around the helical axis of Pep12 is calculated as the degree of rotation between the vector connecting the centers of mass of Pep12 and Get3 at time zero, and at any given point in the trajectory (helical rotation). Color mapping corresponds to the number of snapshots within a given bin, as described in the color scale on the left. Significant clusters within the heat map are highlighted with a green square, and given an index. (**b**) Heat map of the 2-D frequency analysis for the Pep12-Cr-ArsA2 simulation. Axis, color mapping, and cluster indexing are the same as in (**a**). (**c**) Heat map of the 2-D frequency analysis for the Cr-sec61β-Cr-ArsA2 simulation. Axis, color mapping, and cluster indexing are the same as in (**a**). (d) Average structures calculated from indexed clusters of the Pep12-Cr-ArsA2 simulations. Numbers on the top right correlate with the highlighted areas in Fig. 4b. 1. Initial state, corresponding to helical rotation 0, and which is very similar in all structures. 2. Intermediate state, as the protein slowly rotates around Pep12. 3. Highest populated shell, showing low hydration, and high helical rotation. 4. similar state to 3, where the binding groove is open, allowing for easier solvent access. Pep12 is shown in red, while each Cr-ArsA2 chain in green and blue. For comparison purposes, 1. is shown as a shadow in 2. to 4. All structures were aligned to the Pep12 backbone. Helices are numbered according to the Get3 numbering^25^.

Once we could establish that Cr-ArsA2 was indeed a TA-binding protein, we decided to take advantage of the recently published Get3 structure^18^,^25^ to generate a closed state-Cr-ArsA2 model, which we could then compare to other TRC40 like proteins. The resulting 3-D model is a homodimer, which contains most of the Cr-ArsA2 sequence (residue 6 to 357). All features present in Get3, such as two ATPase motifs, and an α-helical hydrophobic groove spanning both monomers, are present. However, since the conserved CXXC motif is absent from Cr-ArsA2 enzyme, a zinc-cysteine coordination site is not present in our model. Limiting the utility of our model is the fact that, in all published TRC40-TA complexes, there is no structural information for the solvent exposed face of the binding groove, where the TRC40-insert is. However, there is strong evidence that the TRC40-insert may serve as a ‘lid’ to help prevent TA exposure to solvent during targeting, and assist TA substrate insertion into the endoplasmic reticulum (ER) membrane^18^,^25^. By modeling the regions as disordered loops, followed by MD simulations, we addressed this issue. On the one hand, we generated several high quality models for Cr-ArsA2 in complex with TA protein Pep12, which we could then compare (Fig. 4, Video S1). On the other hand, by subjecting Get3 to the same process, we had a biochemically and structurally very well characterized system, which we used to validate our bio-computational results.

In our trajectory, Get3-Pep12 rapidly converges to a stable conformation (Fig. S5a), which is very similar to the crystal structure (19.5 ns, backbone RMSD 2.43 Å, Fig. 4a). Cr-ArsA2-Pep12 converged slowly, and towards a more distinct conformation (30.2 ns, backbone RMSD 2.9 Å, Fig. S5a). A complex rearrangement caused the protein to rotate along the Pep12 longitudinal axis during the first 60 ns (helical rotation, Fig. S5b), ceasing at 38.44 + /−4.53 degrees (Fig. 4b, and video S1). Simultaneously, the Cr-ArsA2 groove closed around Pep12, as visualized by the hydration shells of the TA termini, to a final status which resembles Get3 hydration levels (Fig. 4). While Get3 presents a single population, Cr-ArsA2 shows four population clusters (Fig. 4). Cluster one corresponds to the initial stages of the simulation (2.4 to 6.1 ns), cluster two to the rearrangement period (7.7 to 52.9 ns), and cluster three and four are within the rearranged segment of the trajectory, where full rotation has occurred (Figs. 3b, 61.1 to 158.1 ns, and 56.7 to 159.7 ns, respectively, in Fig. S5b). The most highly populated region is cluster three, with low hydration and high rotation (31.28% of the total trajectory, Fig. 4b and d). Behind the Pep12 dehydration lies helix 8 within the TRC40-insert. Helix 8 has been experimentally described by Mateja *et al*.^25^ for the Get3 system, and has been proposed to be a key component to the ‘lid’ effect of the TRC40-insert. Although in our ArsA2 model we modeled the corresponding amino-acids as disordered loops, the helix was formed *in situ* in the trajectory. As helix 8 formed, it closed down on the bound Pep12, displacing the terminal hydration shell of the ligand (Fig. 4). In a strikingly similar series of events to that proposed recently by Mateja *et al*.^25^, the simulation resulted in an equivalent conformation to the published Get3 structure, further cementing the quality of our model. The low hydration state co-exists with a minor population of an open, yet rotated conformation (cluster four, Fig. 4b and d), suggesting that transient groove opening plays an important role in allowing for optimal Cr-ArsA2-Pep12 interactions. Binding enthalpy analysis revealed that at first, Cr-ArsA2 was a poorer Pep12 binder (134 kCal/mol), when compared to the final stages (153 kCal/mol for cluster 3). Crucially, Get3’s single peak yielded a binding enthalpy of −153 kCal/mol, indicating that our Cr-ArsA2 model is a good Pep12 binder.

**Figure 5:**
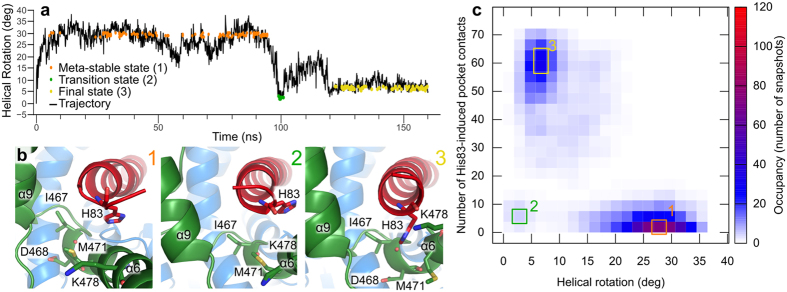
Analysis of the Cr-TRC40-Cr-sec61β trajectory. (**a**) Helical rotation time course along the 160 ns simulation. Snapshots pertaining to the highest occupancy bins in Fig. 5c, are highlighted here as colored data points. (**b**) Relative positions of Cr-sec61β-His-83 towards helix 6. 1 corresponds to the meta-stable (left), initial state, 2 to the short lived transitional state (center). 3 shows His-83 inserted into the induced pocket in the final state. All structures were produced as average coordinates based on the highest occupancy shells in the 2-D frequency analysis. Helix numbering is equivalent to Get3^25^. (**c**) 2-D frequency analysis. 1600 snapshots from the Cr-ArsA2-Cr-sec61β trajectory (video S2) were analyzed for their helical rotation, and for the appearance of His-83-induced pocket contacts. Binned data shows three clusters, as highlighted by the colored squares. Color coding corresponds to the number of snapshots within each bin, as shown in the corresponding color scale (right).

We also substituted the yeast Pep12 ligand for *C. reinhardtii* Sec61β (Cr-sec61β, Fig. 4c). In this case, the 160 ns trajectory converges somewhat intermediately between the previous two (25.6 ns, backbone RMSD 2.6 A, Fig. S5a). The rotation angle distribution is narrower, with maximum values below 40 degrees, and a final conformation similar to the initial one (0 ns vs. 118.1 to 160 ns in Fig. 5a). Also, a long-lived, high-torsion, meta-stable state dominates the first part of the trajectory (5.3 ns to 98.7 ns Fig. 5a). Interestingly, His-83, at the C-terminus of Cr-sec61β, undergoes a dramatic transformation during the simulation (Fig. 5b), with the imidazole side-chain ultimately lodging itself in an induced pocket formed between amino-acids Lys-478, Met-471, Asp-468, and Ile-467 in helices 6 and 9 (Fig. 5).

Taken together, here we can see a three stage conformational change (Fig. 5c). First, the protein rapidly rotates around the Cr-sec61β longitudinal axis (5.2 ns, Fig. 5, cluster 1). At 93.5 ns, Cr-ArsA2 rotates back to within 5 degrees of its original position (Fig. 5, cluster 2). After 23.1 ns of fluctuation, the final state follows (Fig. 5, cluster 3) as His-83 reaches its final conformation. Accordingly, interaction energies in cluster 1 are −105 kCal/mol, cluster 2 represents a high-energy meta-stable state, at −85 kCal/mol, while cluster 3 is the most stable, at −114 kCal/mol. The function of His-83 is particular to Cr-sec61β; however, in the Get3-Pep12 complex, a similar function is accomplished by the interaction between Pep12 Phe-283 and a methionine cluster in Get3 (Fig. S6)^25^. As a result, both complexes show very little freedom of rotation around the hydrophobic ligand. Thus, the simulated Cr-ArsA2-Cr-sec61β complex behaves very similarly to Get3-Pep12, further supporting the formers’ function as a Get3-like targeting factor in *C. reinhardtii.*

## Conclusion

Although there are over 400 predicted plant TA proteins^6^, very little is known about their targeting machinery. Here, we have purified and characterized yeast Get3 plant orthologues, Cr-ArsA2 and Cr-ArsA1, which readily oligomerize, and display strong oxyanion-independent ATPase activity. Most notably, both are capable of forming a complex with TA proteins. Furthermore, we showed that Cr-ArsA1 has some specificity for the chloroplast targeted TOC34-NTC domain, providing the molecular basis for Cr-ArsA1 mediated chloroplast transport^33^. In parallel, we also showed that Cr-ArsaA2 prefers ligands targeted for the endoplasmic-reticulum. Based on comparing MD simulations of both ArsA2 and Get3 with the published, experimental data on Get3, we also propose that, during the cytosolic portion of the PTP, TRC40-insert acts as a TA-helix solvent shield.

Overall, the current data confirms the role of Cr-ArsA1 in delivery of integral membrane proteins to the chloroplast^33^, while suggesting a Cr-ArsA2-mediated, plant-specific targeting mechanism for the endoplasmic reticulum. The possibility of subcellular compartment targeting specificity for TRC40-like proteins has broad implications in all aspects of plant biology and biotechnology, and will elicit much research in the field of plant cellular and molecular biology.

## Materials and Methods

### Gene synthesis

The full-length genes of TRC40 homologs and TA proteins, and DNA oligonucleotides were synthesized by Genomics (Taipei, Taiwan). Sequence verification was performed at Mission Biotech (Taiwan).

### Cloning, expression and purification of TRC40 homologs and TA proteins

Full-length open reading frames (ORFs) corresponding to a series of TRC40 homologs were subcloned into a pET21 vector, modified to incorporate a tobacco etch virus (TEV) protease cleavage site between an N-terminal 6His-tag and the polylinker. For Cr-ArsA1 expression, we used glutathione S-transferase (GST) as a fusion tag for enhancing protein production. After confirming constructs by DNA sequencing, Cr-ArsA2 or Cr-ArsA1 were expressed in *E. coli* BL21 (DE3). Cells were first grown at 37 °C until they reached an OD_600_ of ~0.6, at which point the culture was induced with 0.5 mM isopropyl β-D- 1-thiogalactopyranoside (IPTG), and cultivated at 22 °C for further 20 hours. Cells were disrupted in the presence of protease inhibitors using a high-pressure microfluidizer. After clearing by centrifugation, the supernatant was batch purified by Ni-NTA affinity chromatography. This was followed optionally by cleavage with 6xHis-tagged TEV protease and removal of residual uncleaved target protein and 6xHis-tagged TEV protease by subtractive Ni-NTA purification. The cleaved material was concentrated and then purified by size-exclusion chromatography using a Superdex-200-16/600GL column (GE Healthcare) on a Bio Logic DuoFlow LC system (BioRad). This strategy typically resulted in multi-milligram quantities of highly purified, soluble protein for Cr-ArsA2 and Cr-ArsA1. We regularly estimated protein concentration by A280 using calculated extinction coefficients^39^. For co-expression, *E. coli* BL21 (DE3) was co-transformed with the pET21 vector carrying native Cr-ArsA2 or Cr-ArsA1 expression casettes, and with pET28 containing either an expression cassette for C-terminally His-tagged Sec61β, or one for the NTC region of TOC34 (TOC34-NTC)^40^. TOC34-NTC contains the predicted TMD domain, 12 N-terminal amino acids, plus the remaining 52 C-terminal amino acids, called the hydrophilic C-terminal sequence (CTS)^40^. According to previous experiments by Dhanoa *et al*., NTC stands for N-terminal, TMD, CTS region, and has the following sequence, which was also employed here (TMD is underlined): HPRLSSKPSHRFRWLLPVAIAAEVLFYRRFLH PRLDDNQRRVEREEERVWALRGQQRRALGLHRPHRPDKDAAWRLEQMYDDD^40^. In their publication, Dhanoa *et al*. reported that the up- and downstream segments around the TMD of TOC34 are important for its targeting and translocation. Finally, expression was carried out at 22 °C for ~20 hours by induction with 0.1 mM IPTG after the cells reached an OD600 of ~0.5.

### Liquid chromatography-mass spectrometry (LC-MS) analysis

The mass spectrometry analysis was performed at Mission Biotech (Taiwan). The dried gel pieces containing purified target proteins were prepared for trypsin digestion, and then the peptide mixtures were purified and desalted for mass analysis. LC-MS/MS analysis was performed on an ABI 4700 TOF–TOF Proteomics Analyzer (Applied Biosystems). Data were searched using GPS Explorer (V3.6) with the search engine MASCOT.

### ATPase activity assays

The ATPase activity of Cr-ArsA2 and Cr-ArsA1 was measured using a high-throughput assay in which ATP hydrolysis is coupled to the oxidation of NADH^41^. Because NADH absorbs strongly at 340 nm, but NAD^+^ does not, the decrease in NADH concentration can be monitored spectro-photometrically. Reactions were initiated by adding MgCl_2_ and monitored continuously at 30 °C using a microplate photometric assay. The assay buffer contained 50 mM Tris, pH 7.5, 20 mM NaCl, 5 mM MgCl_2_, 1 mM DTT, 4.5 mM phosphoenolpyruvate, 8.0 U lactate dehydrogenase (Sigma), 6.3 U pyruvate kinase (Sigma), 0.3 mM NADH and 2 μM enzyme. Reactions were carried out in a final reaction volume of 200 ml. The Vmax of the ATPase activity was about 400/nmols/min/mg of Cr-ArsA1 and 480/nmols/min/mg of Cr-ArsA2.

### Construction of the Cr-ArsA2 model

The structural model for Cr-ArsA2 was made using homology modeling procedures based on multiple alignment of the proteins from Get3 family, including the known three dimensional structure of yeast *Saccharomyces cerevisiae* Get3 (Protein Data Bank ID: 2WOJ, nucleotide ADP-ALF4- bound Get3). The 3-D structural model for Cr-ArsA2 and further energy minimization procedures were performed via Discovery Studio 2.5 software of Accelerys (San Diego, CA, USA).

### Molecular dynamics

All simulations were performed with the AMBER14 package^42^, using the amber ff14SB force field^43^, TIP3P water model, and the corresponding monovalent ion parameters^44^.

### File preparation

A PDB file corresponding to the Get3 crystal structure (PDBID #4XTR) was first manually edited to remove extra chains, waters, and other ligands beyond the yeast TA protein Pep12-transmembrane helix. In order to generate Cr-ArsA2-transmembrane helix complexes, the previous file was aligned to a discovery studio generated Cr-ArsA2 model using pymol^45^ with the Cr-ArsA2 and Pep12 helix saved as a complex file. The *C. reinhardtii* Sec61β (Cr-sec61β) sequence was obtained from previously published data, and generated by in-silico mutagenesis in coot^46^, using Pep12 as a template. Finally, coot was employed again to manually add any missing loops in all structures. After the protonation state of the structures were predicted via the H++ protonation server^47^, files were input into tleap to produce initial coordinates and topologies. All complexes were neutralized with sodium ions, while ten extra sodium and ten chloride ions were added to account for a moderate ionic strength.

### Minimization

Minimization was performed in four steps. First, solute atoms were constrained with a 500 kcal/mol harmonic restraint. SHAKE-restrained^48^ water and ions were allowed to relax for 500 cycles of steepest descent, followed by 4500 steps of conjugate gradient. Next, the restraints were lifted from the modeled loops, which were then subjected to 500 cycles of steepest descent and 4500 steps of conjugate gradient. In a third step, restraints on ions, water, loops, and Pep12 or Sec61β ligand were all lifted, and the molecules were allowed to relax under the same conditions as before. Finally, the harmonic restraints on the protein were lifted and the whole system was relaxed for the same amount of cycles.

### Temperature and pressure equilibration

After minimization, temperature was slowly raised over 50 ps from 0 to 300 K, using a Langevin thermostat^49^ (with random seed and γ = 5 ps^−1^), applying weak restraints to the solute molecules. Next, another 50 ps of constant volume simulation at 300 K was performed, in order to further equilibrate the system. Finally, constant pressure, restraint-free equilibration to one atmosphere was carried out for 50 ps (Monte-Carlo barostat, pressure relaxation time 2 ps).

### System relaxation and production dynamics

Keeping the same parameters as in the constant pressure equilibration step, the system was allowed to relax for 2 ns. After relaxation, the system was run for a further 158 ns (498 ns in the case of the Cr-ArsA2 + Cr-sec61β complex).

### Simulation evaluation

CPPTRAJ^50^ was used to extract data from the production trajectories, including RMSDs, hydration, distances, angles, and their population binning. Line art in figures was performed with the Qtiplot and gnuplot software. Clusters were identified via a scipy script. MMGBSA^51^ calculations were used to obtain per-cluster interaction energies. Plotting of data was performed with Qtiplot^52^ and gnuplot. Pymol^45^ was also used for structural figure rendering. Movies were produced via the VMD and lightworks programs.

## Additional Information

**How to cite this article**: Maestre-Reyna, M. *et al*. In search of tail-anchored protein machinery in plants: reevaluating the role of arsenite transporters. *Sci. Rep.* **7**, 46022; doi: 10.1038/srep46022 (2017).

**Publisher's note:** Springer Nature remains neutral with regard to jurisdictional claims in published maps and institutional affiliations.

## Supplementary Material

Supplementary Figures

Supplementary Video 1

Supplementary Video 2

## Figures and Tables

**Table 1:** Effect of oxyanions on ATPase activity.

*V*_max_ (nmols/min/mg)					
ATPase	Oxyanions (0 μM)	Sodium arsenite (100 μM)	relative *V*_max_^*a*^	Potassium antimonite (100 μM)	relative *V*_max_^*a*^
Monomeric Cr-ArsA1	400 ± 25	470 ± 31	1.2	418 ± 26	1.05
Dimeric Cr-ArsA2	480 ± 40	470 ± 35	0.98	377 ± 20	0.8
Yeast Get3^*b*^	418 ± 20	ND	ND	ND	ND
Human ASNA-1^*c*^	17 ± 1	31 ± 3	1.8	17	1
*E. coli* ArsA^*d*^	0.06	0.24	4	1.91	32

^a^*V*_max_ relative to that of without oxyanions.

^b^Kinetic constants obtained from Mateja *et al*.^18^.

^c^Kinetic constants obtained from Kurdi-Haidar *et al*.^36^.

^d^Kinetic constants obtained from Hsu *et al*.^34^.

ND, the experiment was not done.
