# Supplementary material for: In search of tail-anchored protein machinery in plants: reevaluating the role of arsenite transporters
Source: Sci Rep. 2017 Apr 6;7:46022. doi: 10.1038/srep46022 (PMC5382584; doi:10.1038/srep46022)
Supplement: Supplementary Figures [file srep46022-s1.doc]

**In search of tail-anchored protein machinery in plants: reevaluating the role of arsenite transporters**

Manuel Maestre-Reyna^1#^, Shu-Mei Wu^2#^, Yu-Ching Chang^2^, Chi-Chih Chen^2,5,6^, Alvaro Maestre-Reyna^3^, Andrew H.-J. Wang^1,7,8*^, Hsin-Yang Chang^2,4,5*^

1 Institute of Biological Chemistry, Academia Sinica, Taipei, Taiwan,

2 Department of Marine Biotechnology and Resources, National Sun Yat-sen University, Kaohsiung, Taiwan

3 Escuela Tecnica Superior de Ingenierios Industriales, Universidad Politecnica de Valencia, Valencia, Spain

4 The Asia-Pacific Ocean Research Center, National Sun Yat-sen University,

Kaohsiung 804, Taiwan

5 Doctoral Degree Program in Marine Biotechnology, National Sun Yat-Sen University, 70 Lien-Hai Road, Kaohsiung 80424, Taiwan

6 Doctoral Degree Program in Marine Biotechnology, Academia Sinica, 128 Academia Road, Section 2, Nankang, Taipei 11529, Taiwan

7 Core Facilities for Protein Structural Analysis, Academia Sinica, Taipei, Taiwan

8 PhD Program for Translational Medicine, College of Medical Science and Technology, Taipei Medical University, Taipei, Taiwan

# These authors contributed equally to this work.

* Corresponding author

E-mail: ahjwang@gate.sinica.edu.tw (AHJW)

E-mail: hychang@mail.nsysu.edu.tw (HYC)

**Supplementary figures:**


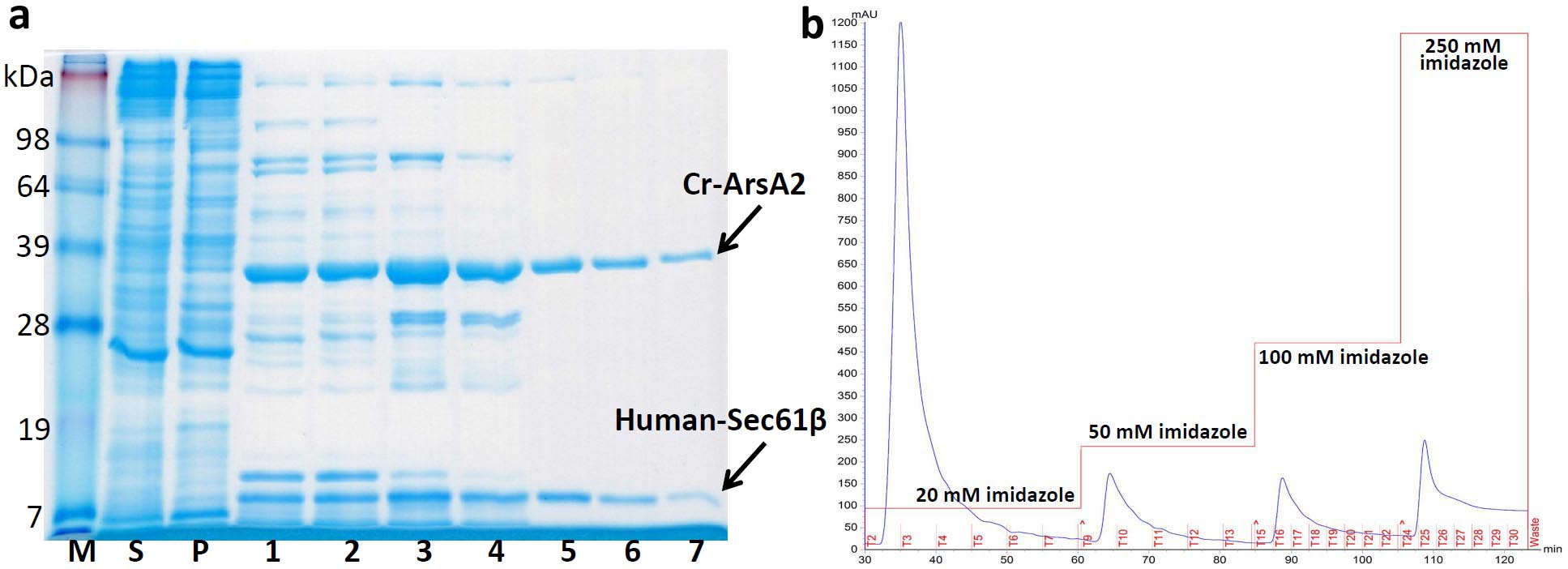


**Fig. S1:** **Cr-TRC40/human Sec61β TA protein complex affinity purified by recombinant co-expression.** FPLC profile of the affinity purification, with SDS-PAGE insert showing lane 1 and 2 – wash with 50 mM imidazole; lane 3 and 4 – wash with 100 mM imidazole; lane 5-7- elution with 250 mM imidazole. S, supernatant; P, pellet; F, flow through. Arrows indicate Cr-TRC40 and human Sec61β with a C-terminal His-tag.

**
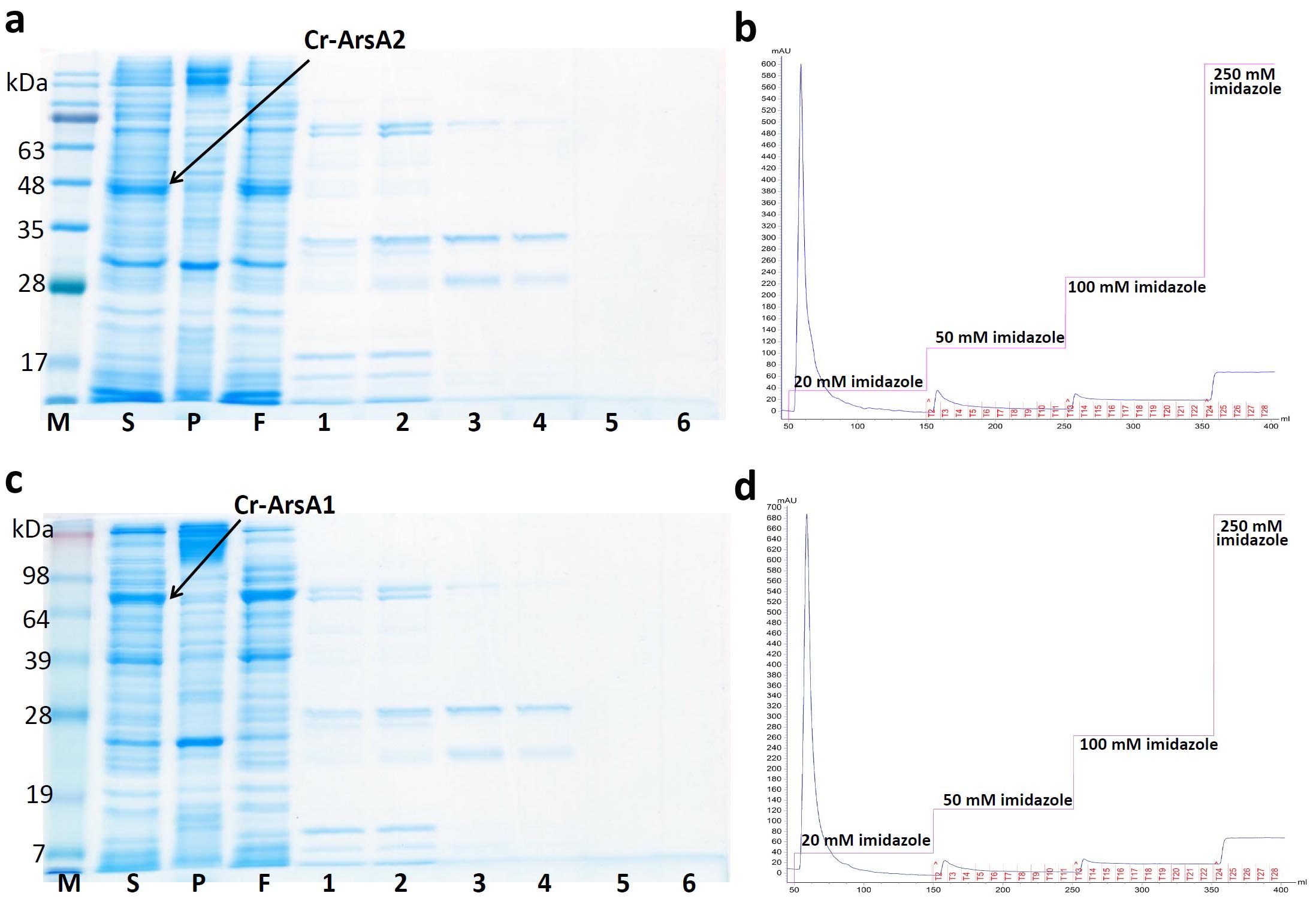
**

**Fig. S2:** **Negative controls for protein complex affinity purified by recombinant co-expression.** (a) and (b) The co-expression and purification of Cr-ArsA2 with a Cr-sec61β containing a C-terminal His-tag but deleted of its TMD. (c) and (d) The expression and purification of native Cr-ArsA1 with no His-tag. FPLC profile of the affinity purification here is similar to those in Fig. S1.

**
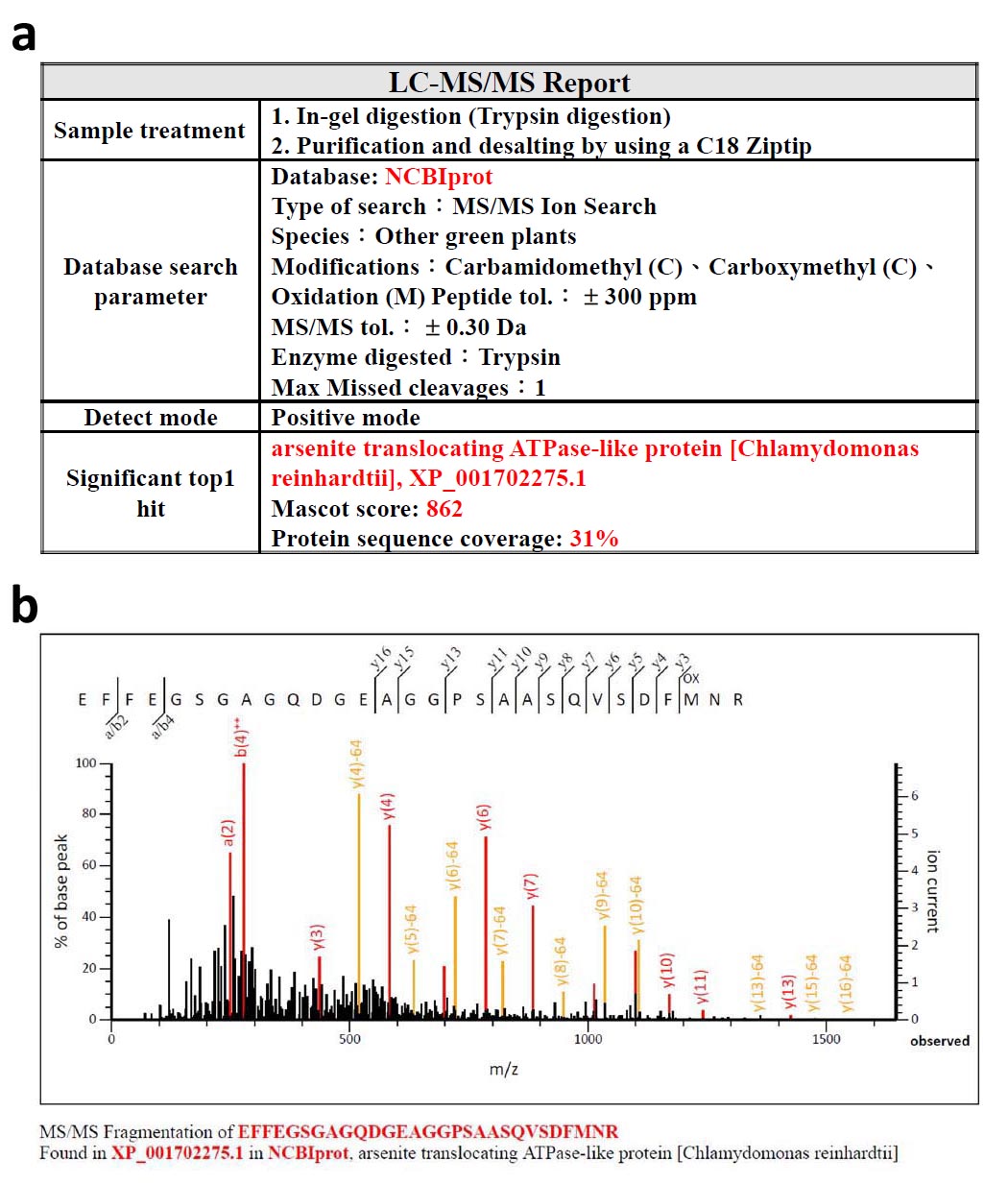
**

**Fig. S3: Liquid chromatography-mass spectrometry analysis.** (a) The major band, marked with Cr-ArsA1 from lane 5 or 6 on SDS-PAGE (Fig. 3c), was excised and subjected to mass spectrometry analysis. The result shows that the significant top one hit is Cr-ArsA1 with mascot score of 862 and sequence coverage of 31% of the whole amino acid sequence of ArsA1. (b) Mascot Peptide view of the MS/MS fragmentation of sequence EFFEGSGAGQDGEAGGPSAASQVSDFMNR is found in Cr-ArsA1.


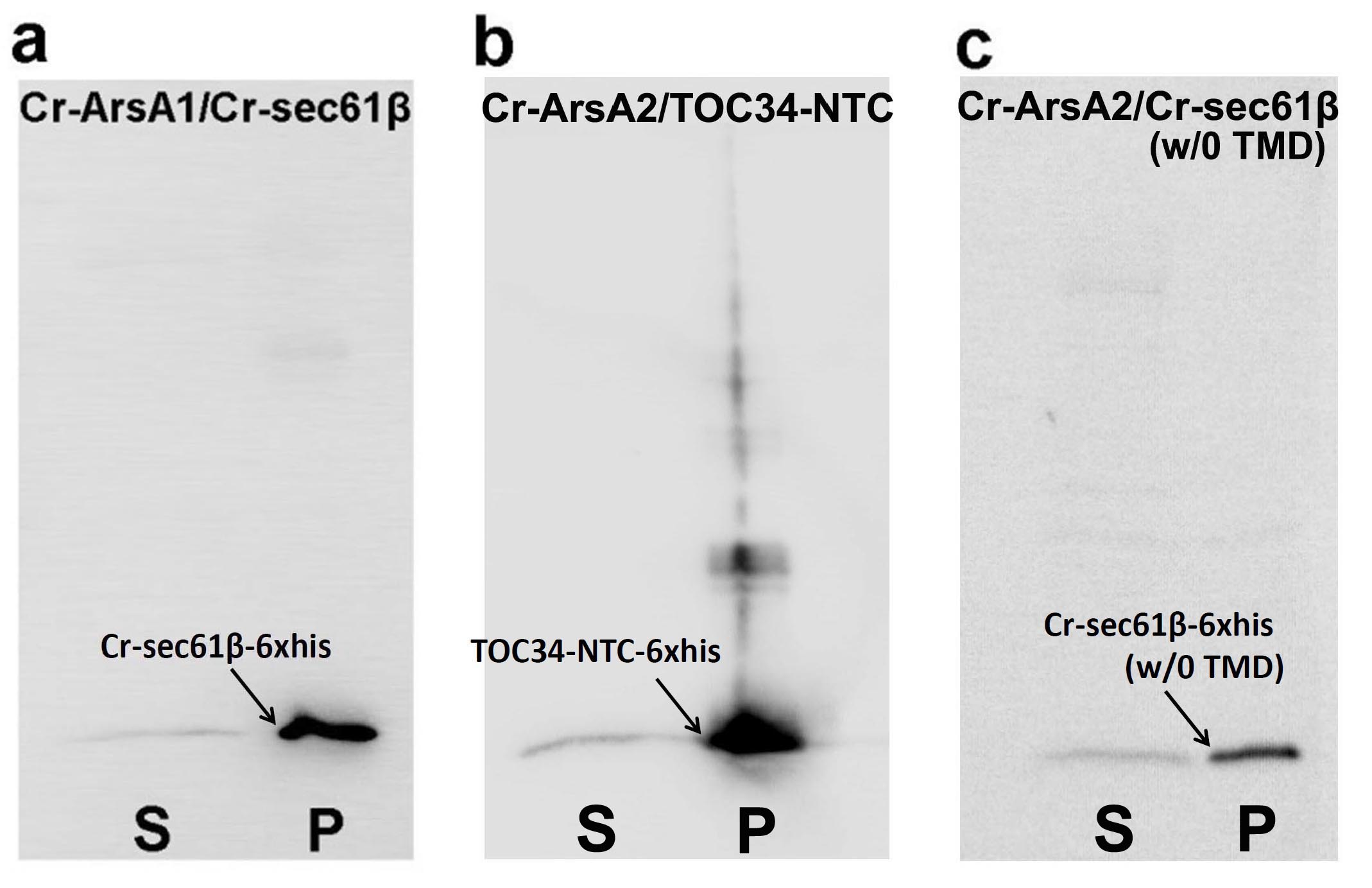


**Figure S4. Western blot analysis of the reverse co-expression of ArsA proteins and TA protein or TMD deleted substrates.** (a) The co-expression of Cr-ArsA1 and Cr-sec61β, (b) Cr-ArsA2 and Cr-TOC34-NTC and (c) Cr-ArsA2 and Cr-sec61β deleted of its TMD. S: Supernatant P: Pellet.

**
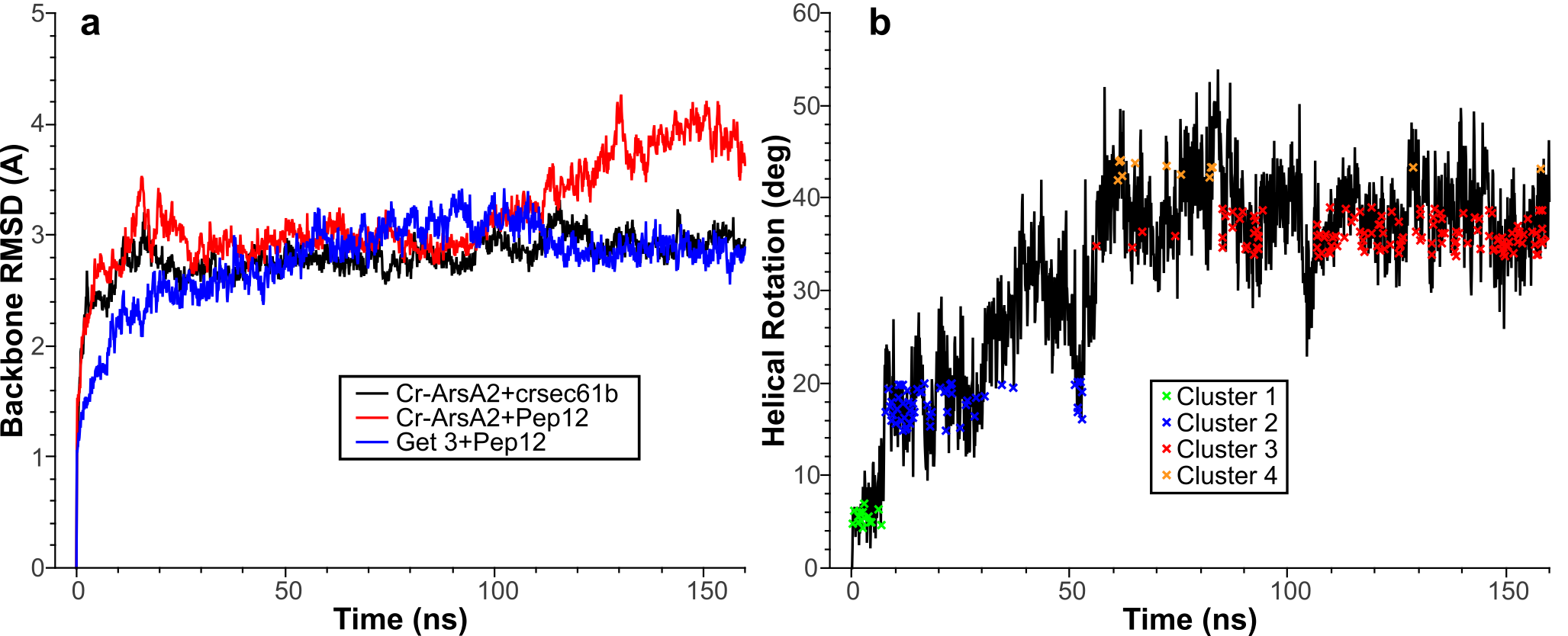
**

**Fig. S5:** **Molecular dynamics time-dependent trajectories.** (a) Backbone Root Mean Square Deviations (RMSD) over time for the Get3+Pep12 (blue), Cr-ArsA2+ Pep12 (red), and Cr-ArsA2+ Cr-sec61β (black). (b) Helical rotation trajectory for the Cr-ArsA2 + Pep12 simulation. The location of clusters one through four in the trajectory, as described in the main text, and in figure 4, are highlighted as colored crosses. Each cross corresponds to a snapshot occupying the highest populated bin of the cluster.


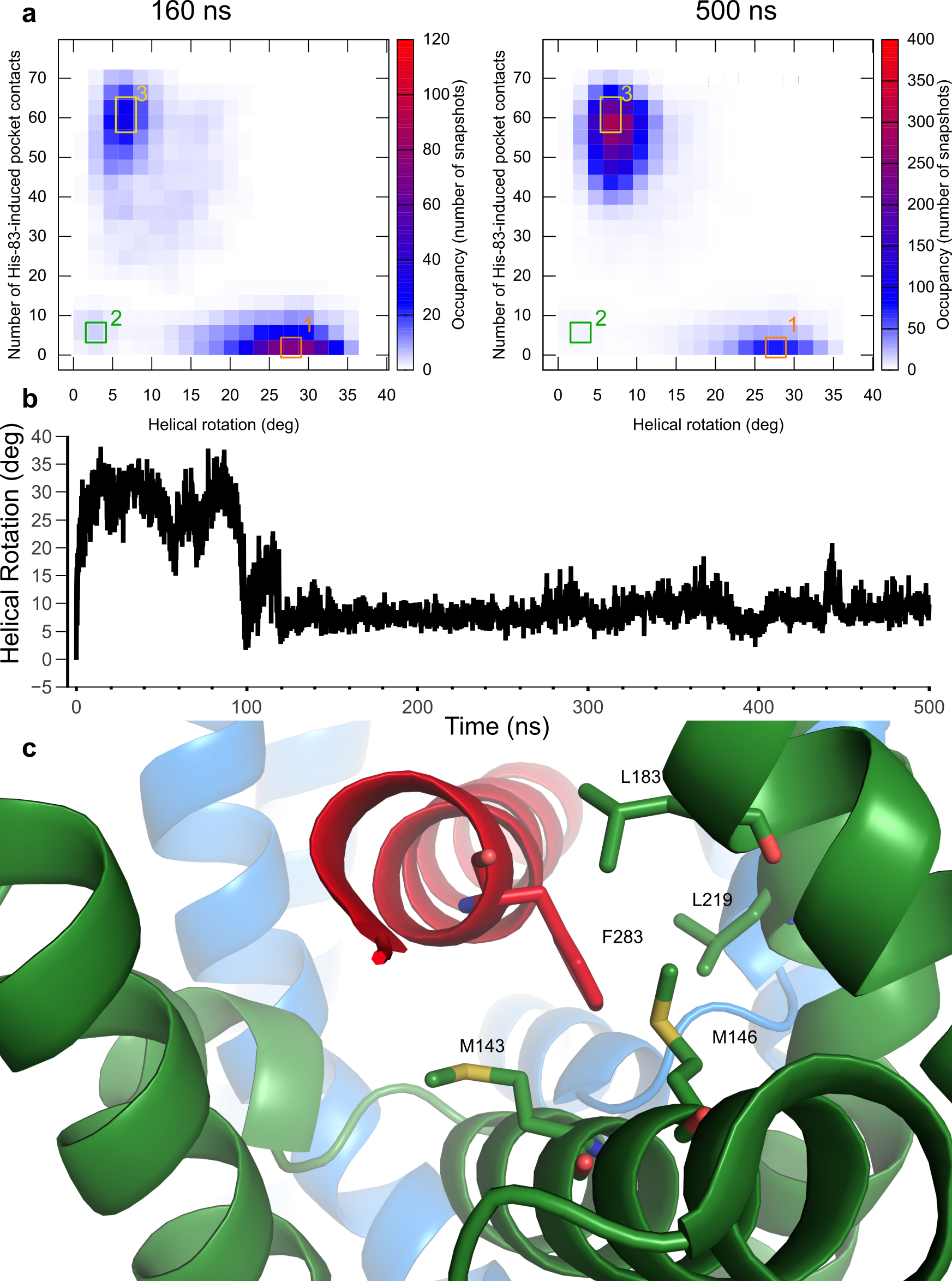


Fig. S6: Prolonging the Cr-ArsA2+Cr-sec61β MD simulation. The final stage of the 160 ns Cr-ArsA2+Crsec61β MD simulation presented an important conformational change, which resulted from the insertion of Crsec61 β His-83 into Cr-ArsA2. In order to confirm that this conformation had much reduced conformational freedom, we prolonged the simulation to 500ns. (a) Comparison between the 160 and 500 ns frequency analysis. The same clusters are present in both histograms. However, cluster 3, corresponding to the final conformation in the short simulation, has become much more prominent in the long one. (b) Helical rotation trajectory for the 500 ns simulation. After the cluster 3 conformation has been adopted (116.6 ns), the complex is locked for the rest of the simulation. (c) Get3 helical rotation is also locked via interaction of Phe-283 in the ligand, and four amino-acids within the binding groove.


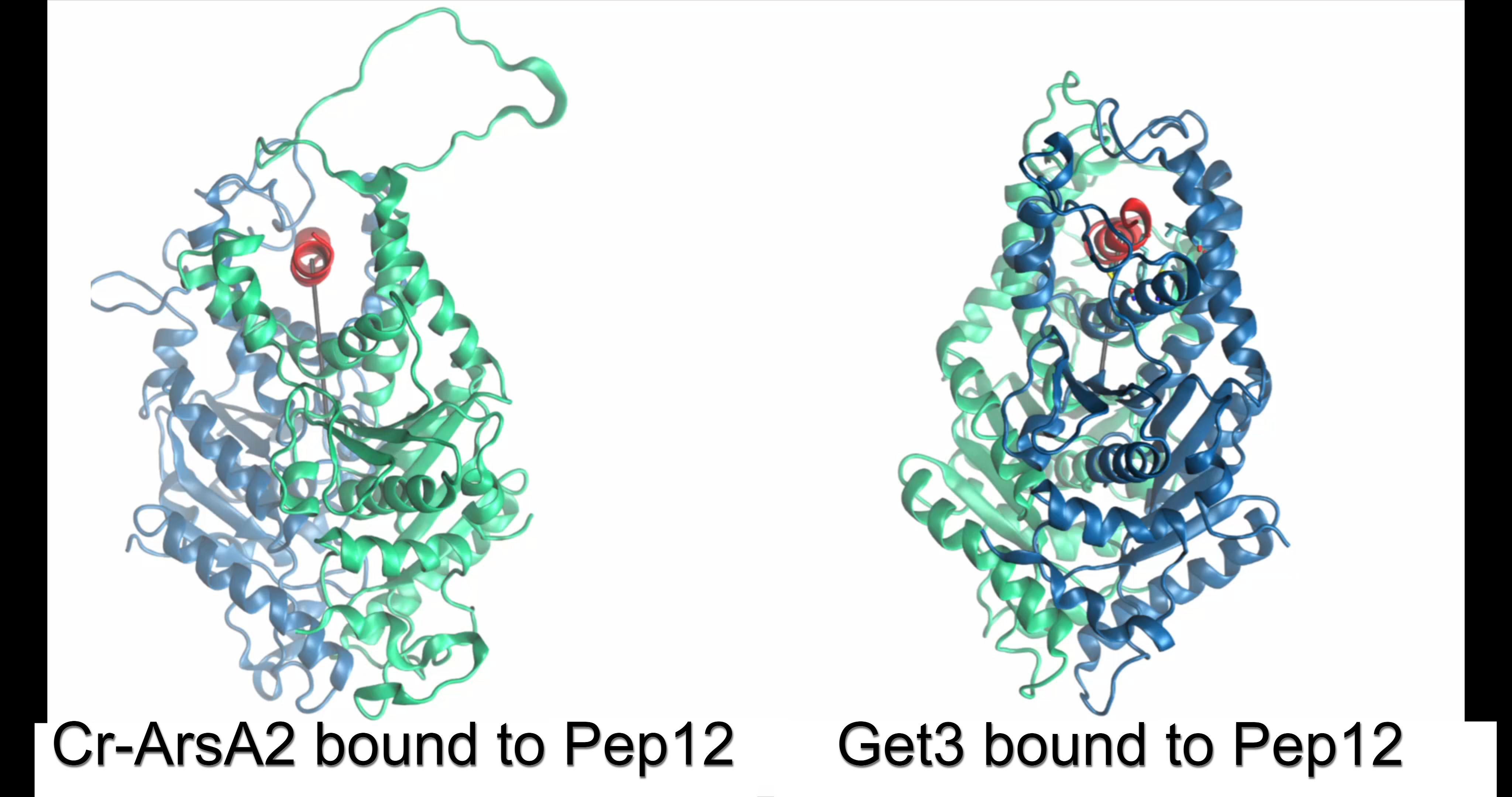

Fig. S7 Supplementary Video S1: Behavior of Cr-ArsA2 bound to Pep12 (left) compared to the Get3-Pep12 complex (right). The Phe-283 binding pocket described in supplementary figure two is explicitly shown here as stick models of the Get3 simulation. The vector employed for determining helical rotation is presented here as a thick grey line. Protein chains are shown in green and blue, while the TA ligand in red.


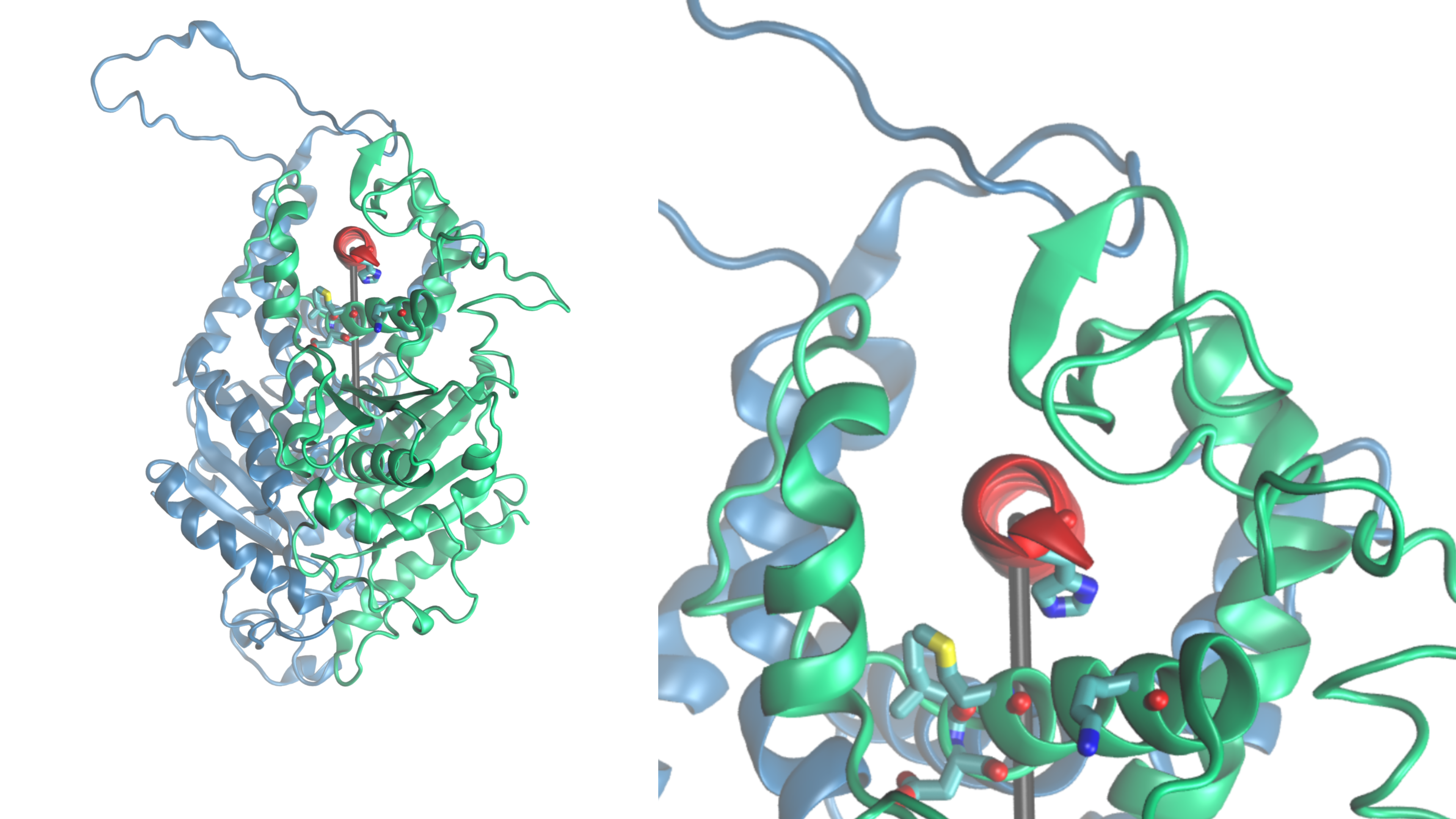

Fig. S8 Supplementary Video S2: behavior of the Cr-ArsA2-Cr-sec61β complex. Left: view of the full protein. Right: Detail of the binding pocket, highlighting the conformational change that leads to an induced pocket for His-83, as shown in Fig. 4. Cr-ArsA2 chains are shown as green/blue cartoons, while the Cr-sec61β ligand as a red cartoon. Finally, protein side-chains involved in the conformational change are shown as stick models. Protein chains are shown in green and blue, while the TA ligand in red.
